# Tobacco consummation: Is it still a dilemma?

**DOI:** 10.4103/1817-1737.69103

**Published:** 2010

**Authors:** Mohamed S Al Moamary

**Affiliations:** *College of Medicine, King Saud bin Abdulaziz University for Health Sciences, Riyadh, Saudi Arabia*

Tobacco consumption is an ongoing public and health problem worldwide. Tobacco is known to play a significant role in many diseases leading to either death or disability. This is not a surprise as there are more than 4000 chemicals and toxins present in inhaled cigarettes. Recently, water-pipe smoking has emerged as a health risk that did not receive enough attention.[1] In Saudi Arabia, tobacco consummation has been reported to be up to 34%.[2–4] In the developing countries, A report from the World Health Organization (WHO) showed that the consumption is rising by almost 3.4% annually.[5,6] Data extracted from a national survey in Saudi Arabia between 1990 and 1993 showed that the overall prevalence of current smoking was 21.1% for males and 0.9% for females. Most of the smokers were middle aged (21–50).[7] In a rural area in Saudi Arabia, the smoking prevalence was found to be 34.4% and 16.4% for current smokers and ex-smokers, respectively.[8] A survey for the prevalence of smoking among the parents of 1482 schoolboys has identified that the overall rate of smoking was 18% (32% for fathers and 4% for mothers).[9] Recent data published by the WHO in 2008 showed that the overall smoking prevalence was 22%, among male adults was 37%, and among female adults was 6%.[10] These worrying numbers emphasis two major facts: the increasing prevalence of smoking and more women started to smoke over the past two decades. In contrast to Saudi Arabia, tobacco consumption in the United State of America was estimated to be 21% and has dropped to almost half over the past three decades.[11]

Another major concern is the fact that tobacco consumption is prevalent among healthcare professionals and students. A recent study has estimated the prevalence of smoking among medical students was 19% where most of them were aware of health problems related to tobacco.[12] Another study showed that 5.3% were ex-smokers and 13% of male medical students were current smokers using both water-pipe (Sheesha) and cigarettes smoking.[2] The favourite place for smoking was mostly outside the college and their homes.[13] Among students of applied medical sciences, smoking prevalence was found to be 20% in male students and 9% in female students.[14] The above findings showed that tobacco prevalence among educated individuals from colleges of health sciences ranged between 13% and 20%. Based on literature from this country, the prevalence was found to be higher among less educated individuals.[15,16]

Saudi Arabia imposed a law banning smoking in public places following signing FCTC (Framework Convention on Tobacco Control) in 2004. Despite this banning, Saudi Arabia is ranked fourth in the world of tobacco import. The presented data raise a major concern about the hidden health problems related to tobacco consumption. Although there are several papers pertaining to smoking, data on the prevalence of tobacco-related diseases are scanty. The high tobacco consumption in Saudi Arabia would indirectly project the magnitude of COPD, lung cancer, cardiovascular diseases, and other related diseases. Re-enforcement of tobacco banning in the country with special attention to younger age groups would lead to reduction of the morbidity and mortality related to tobacco consummation. Moreover, it is highly recommended to conduct comprehensive epidemiological studies on tobacco-related diseases and their impact on the healthcare system.
